# Making chemical & biological protective gloves vapour permeable reduces thermoregulatory strain better than making armour, respirator or overboots permeable

**DOI:** 10.1186/2046-7648-4-S1-A65

**Published:** 2015-09-14

**Authors:** Christie Garson, Michael J Tipton, James R House

**Affiliations:** 1 Extreme Environments Laboratory, Department of Sport and Exercise Science, University of Portsmouth, Portsmouth, UK

## Introduction

Wearing chemical and biological (CB) protective equipment causes thermoregulatory strain by restricting evaporative cooling. We identified [1] that a moisture vapour impermeable (MVIP) body armour liner (BAL) imposed a greater thermal burden than MVIP CB gloves (G), overboots (O) or respirator (R). The previous study progressively and cumulatively removed these MVIP items across 5 conditions when wearing a CB protective suit. This study is a repeat, except that items were removed in isolation and replaced for subsequent conditions to maintain a more uniform thermal load across comparisons. The aims of this study were to quantify the thermal burden imposed by each MVIP item whilst maintaining a high thermal load between conditions to identify the potential benefits if future equipment was made moisture vapour permeable (MVP). A second aim was to determine whether the previous experimental design [1] influenced the thermal burden imposed by each MVIP item. We hypothesised that removal of a MVIP item would reduce heat strain in this order BAL>G>R>O.

## Methods

Following a favourable ethical opinion, 13 males volunteered for this five-condition, repeated measures study, stepping at a light intensity VO_2_ 13.6 mL.kg^-1^.min^-1^), interspersed with 20-minute rest periods in a hot and dry environment (40.5 **°**C and 20% relative humidity) for a maximum of 170 minutes; the last hour being continuous work. Conditions varied in which combinations of MVIP items were worn with a CB suit. In Control (CON) all items were worn, in subsequent conditions, only one item was removed: N_R _(no R), N_BAL _(no BAL), N_G _(no G) and N_OB _(no O). When removed the mass of the item was substituted at the same body site thereby simulating that item 100% MVP but without reducing the metabolic cost of wearing the item.

## Results

Removing G reduced thermoregulatory strain most, as 7 participants completed the full 60 min of stepping in the final work period compared to 1 (CON), 2 (N_OB_), 5 (N_R_) and 5 (N_BAL_). Removing G attenuated the rate of increase in rectal temperature (T_re_) during the final work period compared to CON by 0.37°C.hr^-1 ^(p < 0.001) resulting in a 6% extension to stepping time during the final work period (p < 0.05). Predicted tolerance time (TT) to a T_re _of 40°C (participants stopped when T_re _= 39°C) was extended by 13.3% (p < 0.01). In N_G_, the rate of cooling was augmented in the final rest period with the final change in T_re _lowered by 0.14°C (p < 0.01). The rise in mean body temperature was attenuated from 90 minutes with the greatest attenuation being 0.24°C (p < 0.0001) in N_G_. During N_G _the physiological strain index (PSI) was reduced by 12.7% (p < 0.001). Removing G also reduced RPE during Rest 2 (p < 0.05), final work (p < 0.001) and final rest (p < 0.0001) and improved ratings of thermal comfort during final work (p < 0.01) and rest (p < 0.001). Removing BAL increased sweat evaporation by 10.2%, yet did not extended TT. Removing R improved the PSI by 15.7% (p < 0.05) but did not improve TT. Removing O did little to reduce thermoregulatory strain.

## Conclusion

With the thermal load maintained across conditions, removal of any of the MVIP items reduced the thermal burden with removing G causing the greatest reduction to thermoregulatory strain. This is in contrast to [1] where BAL afforded the biggest benefit when removed. This method rather than [1] offers a better assessment of the contributing burden of protective equipment in human studies. We partly accept our hypothesis; thermal strain was reduced most by removing G, not BAL.
